# Hearing Impairment Affects Dementia Incidence. An Analysis Based on Longitudinal Health Claims Data in Germany

**DOI:** 10.1371/journal.pone.0156876

**Published:** 2016-07-08

**Authors:** Thomas Fritze, Stefan Teipel, Attila Óvári, Ingo Kilimann, Gabriele Witt, Gabriele Doblhammer

**Affiliations:** 1 German Center for Neurodegenerative Diseases (DZNE), Rostock/Greifswald, Germany; 2 Rostock Center for the Study of Demographic Change, Rostock, Germany; 3 Department of Psychosomatic Medicine, University Medicine, Rostock, Germany; 4 Department of Otorhinolaryngology, Head and Neck Surgery, University Medicine Rostock, Rostock, Germany; 5 Institute for Sociology and Demography, University of Rostock, Rostock, Germany; 6 German Center for Neurodegenerative Diseases (DZNE), Bonn, Germany; 7 Max Planck Institute for Demographic Research, Rostock, Germany; Cardiff University, UNITED KINGDOM

## Abstract

Recent research has revealed an association between hearing impairment and dementia. The objective of this study is to determine the effect of hearing impairment on dementia incidence in a longitudinal study, and whether ear, nose, and throat (ENT) specialist care, care level, institutionalization, or depression mediates or moderates this pathway. The present study used a longitudinal sample of 154,783 persons aged 65 and older from claims data of the largest German health insurer; containing 14,602 incident dementia diagnoses between 2006 and 2010. Dementia and hearing impairment diagnoses were defined according to International Classification of Diseases, Tenth Revision, codes. We used a Kaplan Meier estimator and performed Cox proportional hazard models to explore the effect of hearing impairment on dementia incidence, controlling for ENT specialist care, care level, institutionalization, and depression. Gender, age, and comorbidities were controlled for as potential confounders. Patients with bilateral (HR = 1.43, p<0.001) and side-unspecified (HR = 1.20, p<0.001) hearing impairment had higher risks of dementia incidence than patients without hearing impairment. We found no significant effect for unilateral hearing impairment and other diseases of the ear. The effect of hearing impairment was only partly mediated through ENT specialist utilization. Significant interaction between hearing impairment and specialist care, care level, and institutionalization, respectively, indicated moderating effects. We discuss possible explanations for these effects. This study underlines the importance of the association between hearing impairment and dementia. Preserving hearing ability may maintain social participation and may reduce the burden associated with dementia. The particular impact of hearing aid use should be the subject of further investigations, as it offers potential intervention on the pathway to dementia.

## Introduction

### Demographic background

The prevention and treatment of dementia has become a public priority in the course of demographic ageing. Although studies suggest that trends in medical, lifestyle, and societal risk factors of dementia may have led to a reduction in the prevalence of dementia [1], there still will be a worldwide increase in the number of dementia cases with up to 114 million patients in 2050 [2]. According to projections, industrialized countries such as the U.S., with 4.7 million Alzheimer’s disease dementia cases in 2010 and 13.8 million in 2050 [3], or Germany, with roughly one million cases in 2005 and up to about three million in 2050, will experience a significant increase [4].

While no cure of dementia is available at present, the future number of dementia cases may be altered by disease-modifying interventions to prevent or slow down the progression of the disease [5]. Empirical evidence suggests that intervention in the case of hearing impairment may help to decelerate or to reduce the risk of cognitive decline [6, 7]. Numerous prospective studies have demonstrated an association of hearing impairment and dementia. Lin et al. [8] reported that hearing loss is independently associated with accelerated cognitive decline and incident cognitive impairment. A study by Uhlmann et al. [9] revealed an association of hearing impairment and Alzheimer’s dementia in a case-control study supporting the hypothesis that hearing impairment contributes to cognitive dysfunction. Kiely et al. [10] found an association of cognitive impairment and lower levels as well as faster declines in peripheral hearing ability. Valentijn et al. [11] found evidence that a change in auditory acuity predicts change in memory performance. In a previous study based on health claims data, we analyzed the prevalence of hearing impairment and dementia on a regional level and found a significant association. The association was partly independent of potential vascular risk factors and cerebrovascular disease, supporting the specificity of the effect [12].

### Relevance of dementia and hearing impairment

Both diseases have a high relevance for society. The prevalence of dementia increases exponentially with age and doubles every five to six years, from about 2% at age 65 to 30–40% between ages 90 to 100 [4]. Dementia and cognitive impairment are the major causes for the development of long-term impairment in activities of daily living [13–15] and are the main contributing factor to institutionalization in the elderly population [16], emphasizing the need for appropriate service and intervention programs. Dementia is also a burden for family care givers who often show high levels of psychological morbidity [17] and reduced quality of life [18]. Dementia patients have increased mortality compared to non-demented people [19–21].

There is no common definition or measure of hearing impairment and hearing loss. This makes it difficult to compare prevalence estimations [22]. The most common sensory impairment of elderly people is age-related hearing impairment, also known as presbycusis [23], with a prevalence of about 37% between ages 60 and 70 and about 60% for people aged 70 or older [24]. A review by Ciorba et al. [25] summarized that hearing loss can lead to a reduced quality of life, including physical, material, social, and emotional well-being, and the impact depends on the grade of severity of hearing loss [26]. Consequences of hearing loss may be isolation, dependence, frustration, as well as communication disorders. Similarly, a study by Chia et al. [27] revealed that a combined impairment of hearing and vision affect health-related quality of life. Sensory impairment, i.e. impairment of hearing and/or vision, was shown to be predictive of subsequent functional impairment in older persons [28] and increased the risk of cognitive decline [29].

### Relationship of hearing impairment and dementia

Common to both may be social withdrawal, reduced perception, repeated questioning, impaired short-term or reduced working memory, word finding disorders or communication disorders, as well as problems in following a conversation [26, 30–34].

Possible aspects that may explain the association between dementia and hearing impairment have been summarized by Kilimann et al. [30]. First, there are specific diseases, such as rare hereditary diseases, which may lead to hearing and cognitive impairment. Second, as both diseases frequently occur at old age they may be described as comorbidities. Third, hearing impairment may lead to increased social isolation [35, 36], subsequently increasing the risk of dementia onset [37]. Fourth, hearing impairment may confound the results of cognition tests. Fifth, a neurodegeneration may further deteriorate hearing impairment, e.g. by a specific degeneration of the association cortex [38] or by impairment of comprehension [39]. Sixth, there may be a pathophysiologic interaction of hearing impairment and neurodegeneration.

Given the knowledge of the associations between hearing impairment and cognitive decline as well as dementia, Dawes et al. [40] formulated two hypotheses concerning potential pathways. According to the “common cause” hypothesis the association is attributed to common, age-related neurodegenerative mechanisms working in both hearing impairment and cognitive decline. The “cascade” hypothesis describes a phenomenon including a direct effect of long-term deprivation of auditory input on cognition through impoverished input, or indirectly through the effects of hearing loss on social isolation and depression. In a cross-sectional study with 164,770 participants in the UK, the authors found results consistent with the cascade hypothesis, namely an association of hearing aid use with better cognition.

### Aim of the study

After establishing a regional association between hearing impairment and dementia in our previous study [12], the aim now was to explore possible pathways using longitudinal information from German health claims data. It was hypothesized that the presence of a diagnosis of hearing impairment increases the risk for transition to a dementia diagnosis. Like the study by Dawes and colleagues [40], we adjusted for a series of information to reveal whether these mediate or moderate the relationship of hearing impairment and dementia. A mediating effect considers whether the effect of hearing impairment on dementia runs indirectly through a third condition. A moderating effect considers whether the effect of hearing impairment depends on the value of the moderating variable. In particular, we analyzed four variables. We used treatment by an ENT doctor as a specialist for diseases of the ear (E), nose (N), and throat (T) region. As far as this treatment is particularly related to hearing impairment, we hypothesized that a visit to an ENT doctor is associated with adequate treatment and possibly with the use of hearing aids. This may improve hearing ability and, thus, decrease the hearing-mediated risk of dementia. We used care level as an indicator for the general frailty which may moderate the association of hearing impairment and dementia. We included institutionalization and expect a mediating effect. Similarly, we tested whether depression mediates the relationship of hearing impairment and dementia onset. Age, gender, and a series of co-morbidities were controlled for as potential confounders. We presented the results of event-history analysis and discussed possible explanations for the effects we found.

## Materials and Methods

### Data

We used data of the AOK (Allgemeine Ortskrankenkasse), which is the largest health insurance company in Germany. About one third of the German population is insured through the AOK and the proportion increases with age [41]. A random sample of 250,000 insured persons born in or prior to 1954, who had at least one day of insurance coverage by the AOK in the first quarter of 2004, was drawn by the WIdO (Scientific institute of the AOK). Using a unique person ID, these persons were followed quarterly over time between 2004 and 2010, establishing a longitudinal sample. The data include complete records of the inpatient and outpatient treatment that each insured patient received. The observation period starts at the first quarter of 2006 to ensure incident dementia cases. Excluding persons with inconsistent or missing information on date of birth, date of death, and sex, and studying ages 65 and above we arrived at a study sample of 154,784 persons.

Data access was legally approved by the WIdO. The study is based on anonymized administrative claims data that never involved patients directly. Individual patients cannot be identified and the analyses presented do not affect patients whose anonymized records were used.

### Dependent variable: Transition to dementia

Dementia was defined according to the codes of the International Classification of Diseases, 10^th^ Revision (ICD-10): G30, G31.0, G31.82, G23.1, F00, F01, F02, F03, and F05.1. Due to the character of health claims data as a secondary data source, it was not possible to validate dementia diagnoses in face-to-face examinations. In order to avoid a potential overestimation of the true number of cases we applied a validation procedure (see [4] for details). Incident dementia cases were defined as the first occurrence of a valid diagnosis. Furthermore, to avoid confusion between firstly-diagnosed incident cases and prevalent cases with a history of dementia, a period of at least two years without valid dementia diagnoses was warranted. Thus, incident dementia is defined for all persons who did not have a valid dementia diagnosis in 2004 and 2005, and who were diagnosed for the first time between 2006 and 2010.

### Independent variables

For the indicator of hearing impairment we distinguished five categories including diagnoses according to ICD-10: no diseases of the ear and mastoid process; unilateral hearing impairment (H90.1, H90.4, H90.7, and H91.2); bilateral hearing impairment (H90.0, H90.3, H90.6, H91.0, and H91.1); unspecified hearing impairment without information on laterality (H90.2, H90.5, H90.8, H91.8, H91.9, H65.2-H65.4, H66.1-H66.4, H66.9, H80, H80.1, H80.2, H80.8, H80.9, H71, H74, and H74.1-H74.3); all other diseases of the ear and mastoid process, including all diagnoses not related to hearing impairment. In the case of simultaneously occurring diagnoses we took only one of them into account in the following priority order from high to low: bilateral hearing impairment; unilateral hearing impairment; unspecified hearing impairment; all other diseases of the ear and mastoid process.

We took four key variables into account as potential mediators or moderators. We controlled whether the patient ever received treatment by an ENT doctor during the observation period, but only in the presence of at least one of the above-mentioned hearing impairment diagnoses. We controlled whether the person lived in a nursing home, whether the person ever had a diagnosis of depression during the observation period (ICD-10 codes F32 and F33), and for care level. According to the care level, individuals are entitled to benefits or services from the German statutory long-term care insurance. After passing an assessment mainly based on impairments in activities of daily living, applicants are assigned to one of three care levels (considerable need for care = care level 1, severe need for care = 2, and extreme need for care = 3). We then addressed a series of co-morbidities: diabetes mellitus (ICD-10 codes E10–E14); hypertension (I10–I13, I15); hypercholesterolemia (E78.0); cerebrovascular disease (I60–I69, G45, G46, H34.0); cancer (all codes beginning with C); diseases of the heart (I43, I50, I099, I110, I130, I132, I255, I420, I425-I429, P290); chronic lung diseases (J4, J60, J61, I278, I279, J684, J701, J703); rheumatism (M05, M06, M315, M351, M353, M360); paralysis (G81, G82, G041, G114, G801, G082, G830-G839); injuries to the lower extremities (S7-S9, T003, T006, T013, T016, T023, T025, T026, T033, T034, T043, T044, T053-T056, T12, T13, T24, T25, T336-T338, T346-T348, T355, T871); blindness (H54). A variable in the model indicates whether a person received no, one, two, three, four, five, six, or seven or more of these diagnoses during the observation period. We also took into account a tinnitus diagnosis (H93.1). Demographic information contained gender and five-year age-groups beginning at age 65.

### Statistical analysis

We applied methods of event history analysis to analyze the risk of incident dementia. We used the Kaplan-Meier estimator to measure the fraction of subjects living without a dementia diagnosis for a certain amount of time after the year 2006. Cox proportional hazard models were conducted to analyze the determinants for the risk of dementia incidence. Persons were followed until death or censoring by leaving AOK insurance coverage or death. The censoring time was set in the middle of the last observed quarter, and deaths were assumed to have been in the middle of the month of death. We examined direct associations between hearing impairment and dementia incidence in models adjusted for age, gender, co-morbidities, and with and without the four potential mediators or moderators. A mediating effect appears when the following criteria are met: First, hearing impairment affects the risk of dementia incidence. Second, the potential mediator affects dementia onset in a model that controls for hearing impairment. Third, the coefficients of hearing impairment in the latter model decreases when controlled for the potential mediator. A moderating effect is explored by the interaction effect of hearing impairment and the variable of interest.

## Results

### Descriptive results

The 154,783 persons contributed 669,524 person-years and 14,602 incident dementia diagnoses during the period 2006 to 2010 (Table 1). The overall incidence rate (IR), displayed as incident dementia cases per 100 person-years, was 2.18 (95% confidence interval CI = 2.15–2.22). With regard to hearing impairment, the incidence rates were highest for those persons with bilateral hearing impairment (IR = 3.54, CI = 3.42–3.67), followed by those with unspecified hearing impairment (IR = 2.35, CI = 2.25–2.45). The incidence rate of persons with unilateral hearing impairment was 1.44 (CI = 1.23–1.69) while the rate of persons without any diagnosed disease of the ear was 1.92 (CI = 1.88–1.97). Persons who received treatment by an ENT doctor revealed higher incidence rates (IR = 2.33, CI = 2.26–2.39) than those without treatment (IR = 2.11, CI = 2.07–2.15). Those who live in a nursing home (IR = 26.44, CI = 25.46–27.46) had higher incidence rates compared to those who are not institutionalized (IR = 1.81, CI = 1.77–1.84). The incidence rates for persons with depression (IR = 3.37, CI = 3.28–3.46) exceeded the rates for persons without depression (IR = 1.80, CI = 1.77–1.84).

**Table 1:** Dementia incidence rates by key variables.

Variable	Value	Person-years (PY)	Cases	IR	LCI	UCI
Hearing impairment	no	365,464	7035	1.92	1.88	1.97
	unilateral	10,617	153	1.44	1.23	1.69
	bilateral	91,421	3238	3.54	3.42	3.67
	unspecified	93,959	2205	2.35	2.25	2.45
	other diseases of the ear	108,064	1971	1.82	1.75	1.91
Treatment by an ENT doctor	no	444,605	9371	2.11	2.07	2.15
	yes	224,919	5231	2.33	2.26	2.39
Care level	no care level	614,887	7208	1.17	1.15	1.20
	1	35,396	4021	11.36	11.01	11.72
	2	16,279	2739	16.83	16.21	17.47
	3	2962	634	21.41	19.80	23.14
Nursing home	no	659,308	11,901	1.81	1.77	1.84
	yes	10,216	2701	26.44	25.46	27.46
Depression	no	508,545	9174	1.80	1.77	1.84
	yes	160,979	5428	3.37	3.28	3.46
Total		669,524	14,602	2.18	2.15	2.22

Data source: Claims data AOK 2006–2010;

IR: Incidence rate per 100 PY

LCI: 95% lover confidence interval

UCI: 95% upper confidence interval

Dementia incidence rates for covariates presented in S1 Table

**Fig 1:**
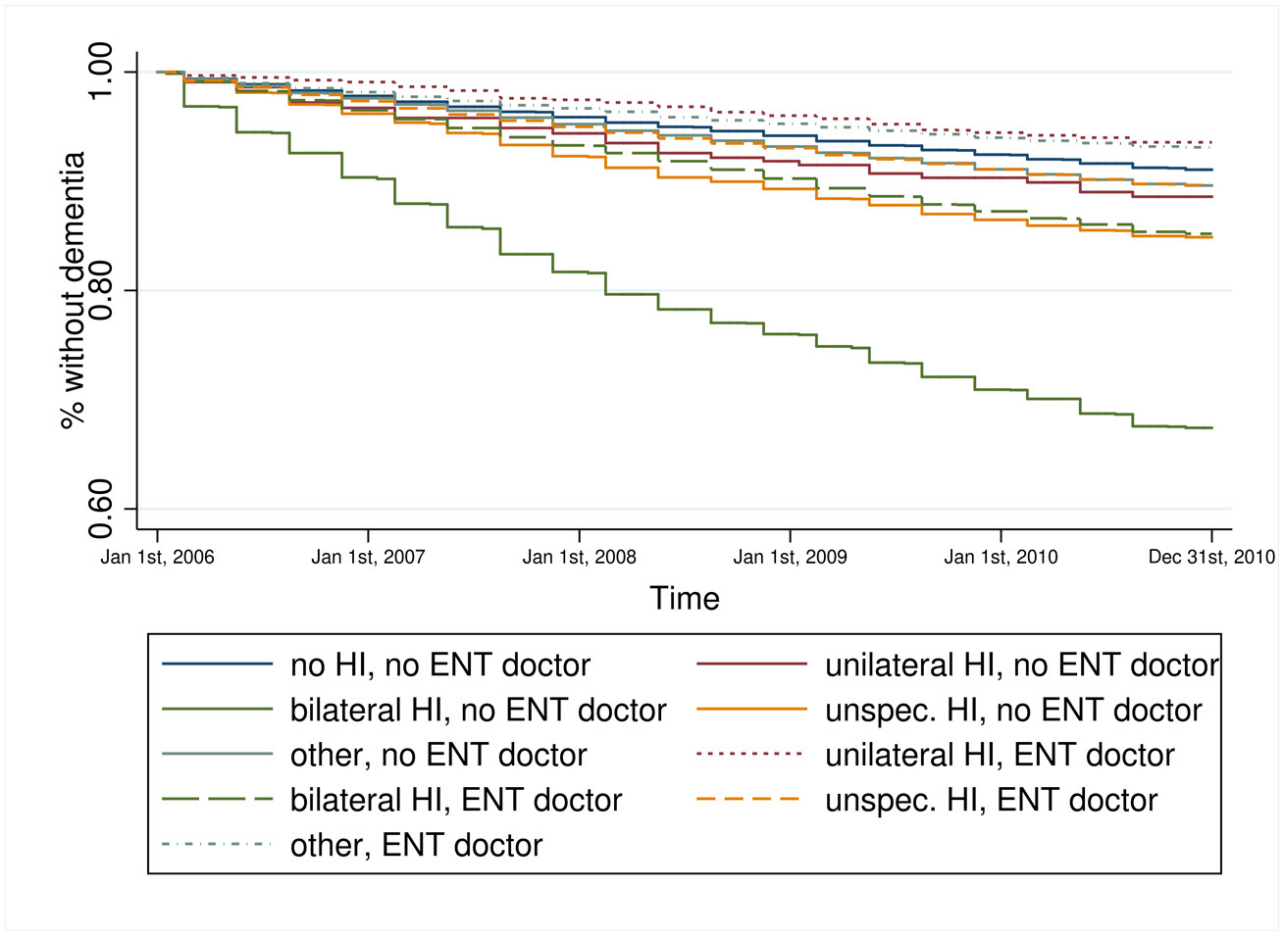
Kaplan-Meier estimator of time to dementia incidence by hearing impairment and treatment by an ENT doctor. Data source: Claims data AOK 2006–2010; p_log rank_<0.001.

**Fig 2:**
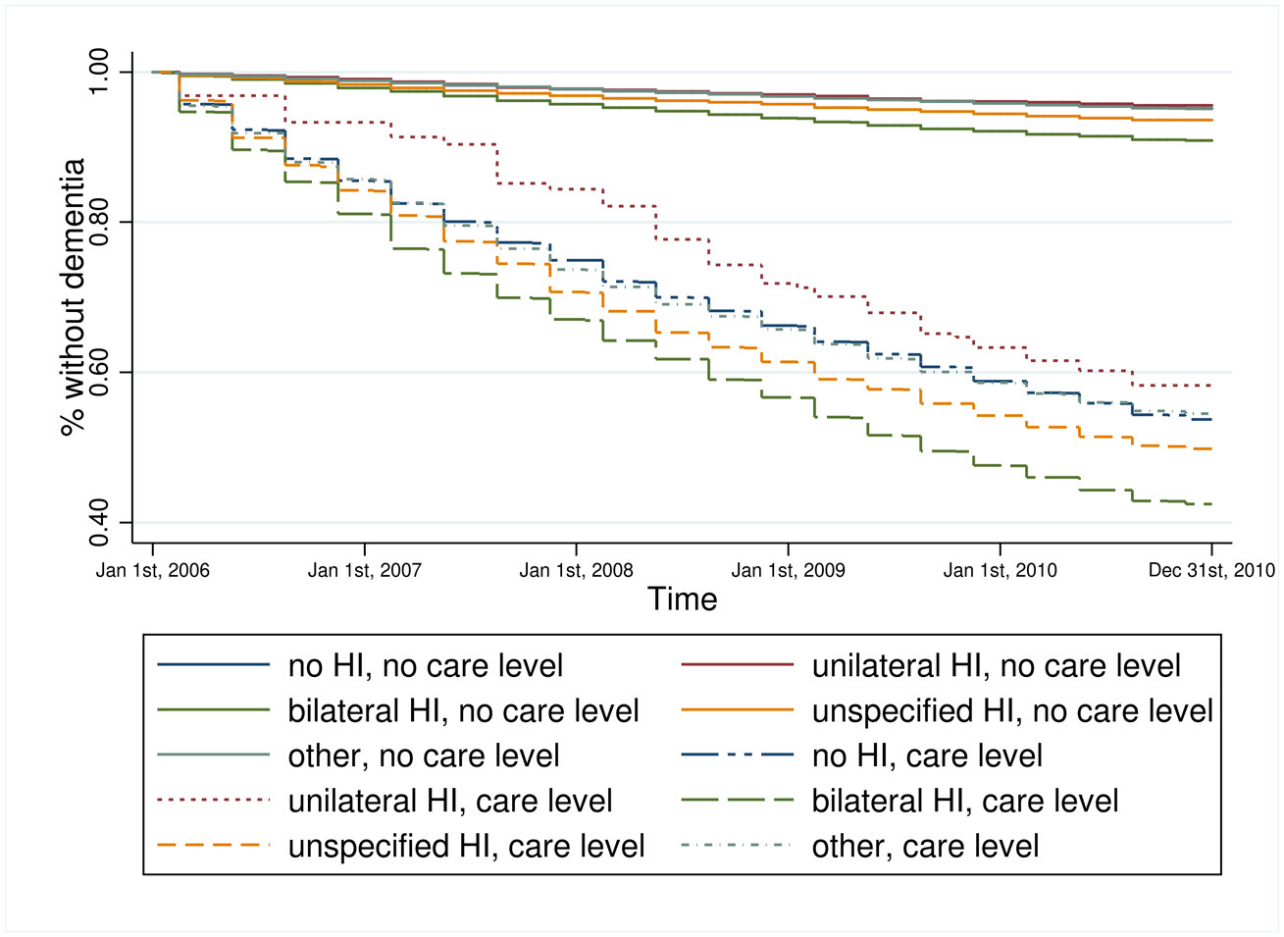
Kaplan-Meier estimator of time to dementia incidence by hearing impairment and care level. Data source: Claims data AOK 2006–2010; p_log rank_<0.001.

**Fig 3:**
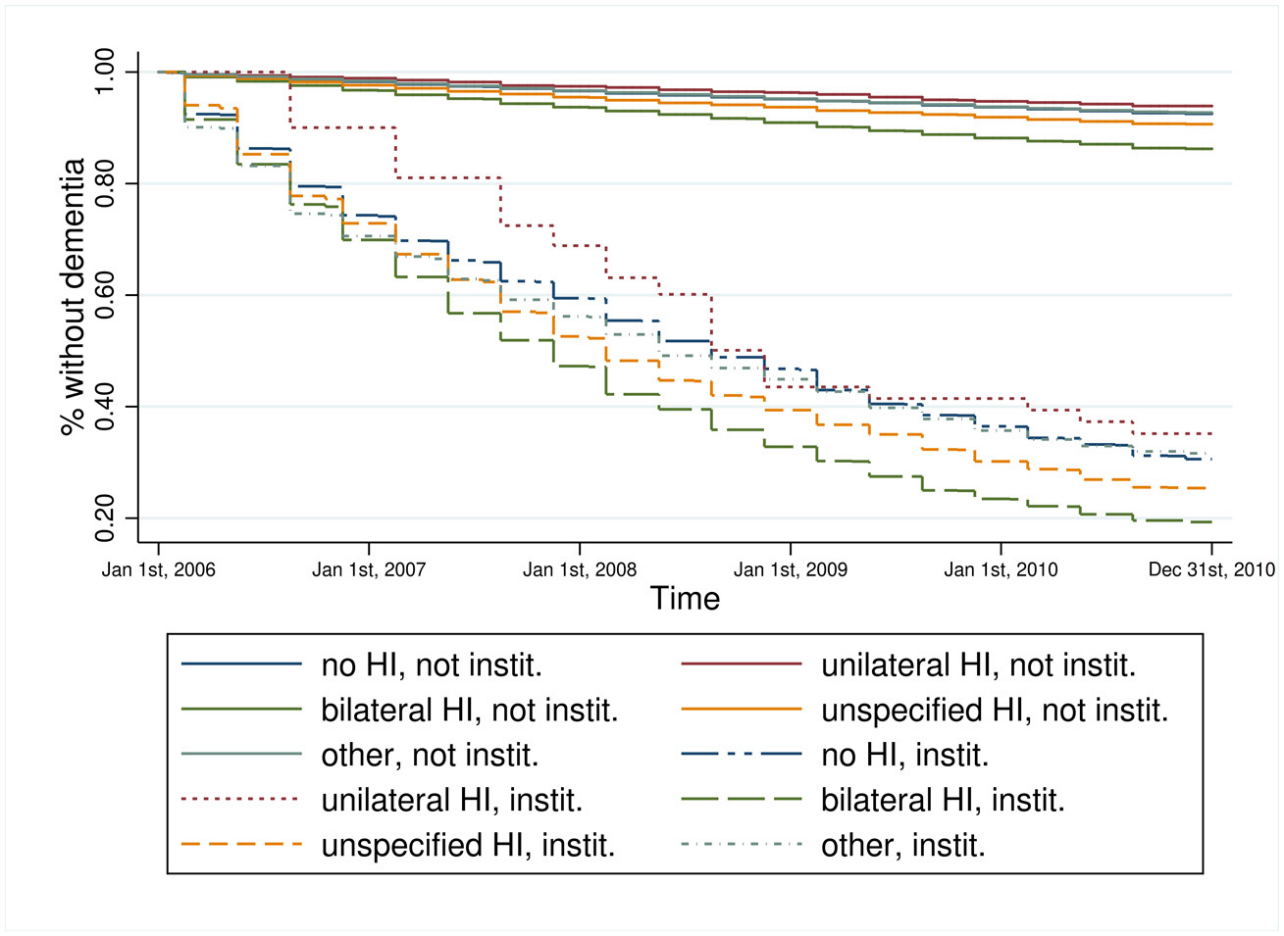
Kaplan-Meier estimator of time to dementia incidence by hearing impairment and living in a nursing home. Data source: Claims data AOK 2006–2010; p_log rank_<0.001.

**Fig 4:**
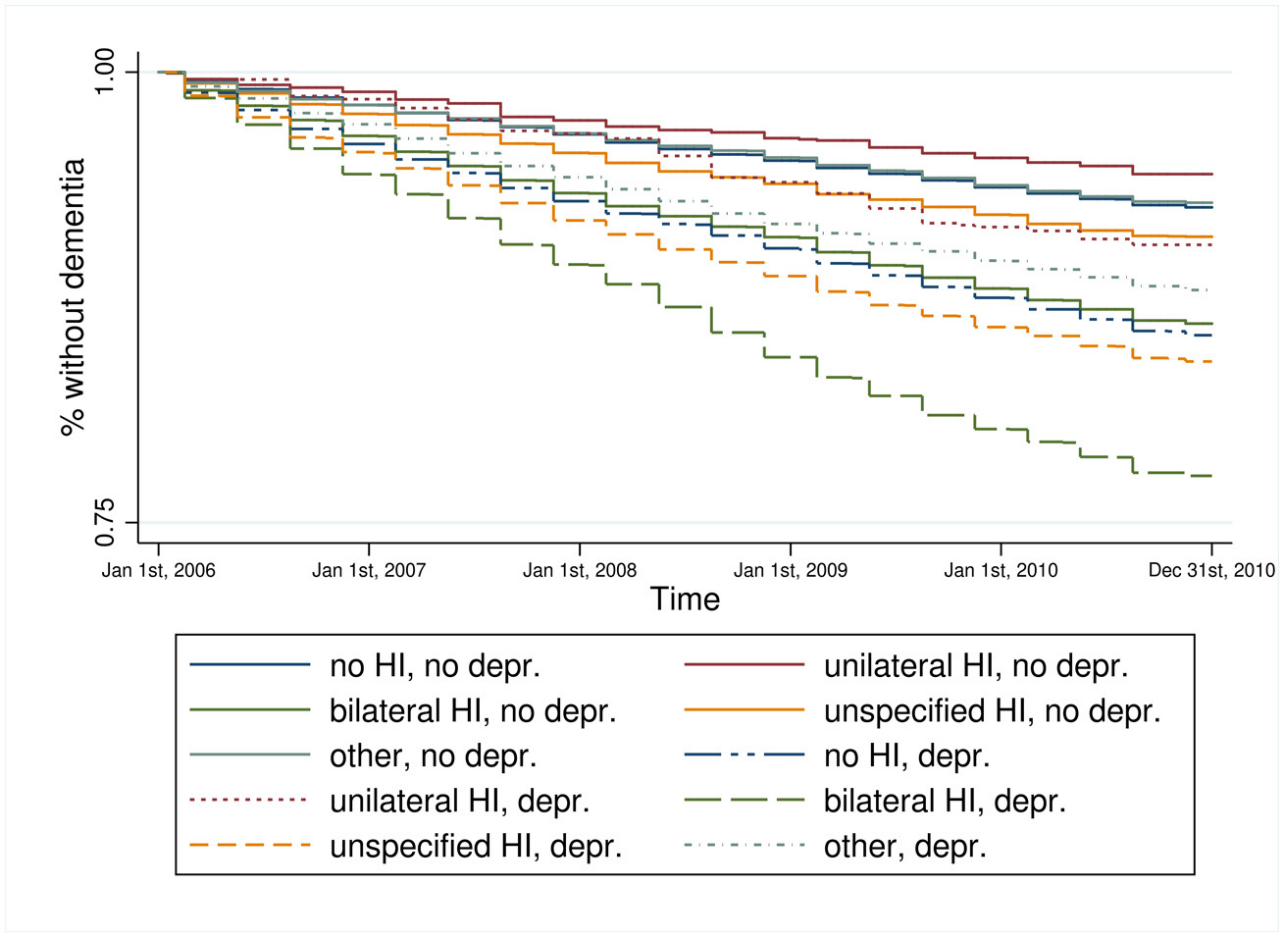
Kaplan-Meier estimator of time to dementia incidence by hearing impairment and depression. Data source: Claims data AOK 2006–2010; p_log rank_<0.001.

Kaplan-Meier estimators for hearing impairment and each key variable on its own (results not shown) revealed that persons with bilateral hearing impairment experienced a faster incident dementia diagnosis compared to all other persons. Persons who received treatment by an ENT doctor experienced a faster incident dementia diagnosis compared to persons without such treatment. The higher the care level the faster the transition to dementia and the same was true for those living in a nursing home. Depression was also associated with a faster transition to dementia. The combined information of hearing impairment and the key variables, respectively, provides a more particular view of the relationship (Figs 1–4). Bilateral hearing impairment without treatment by an ENT doctor led to a faster transition to dementia (Fig 1). For the combination of hearing impairment and care level, we only distinguished between the presence and absence of a care level, not at each of the three levels. Those with bilateral hearing impairment and a care level experienced the fastest incident dementia diagnosis (Fig 2). Similarly, bilateral hearing impairment and institutionalization in a nursing home (Fig 3) as well as bilateral hearing impairment and depression (Fig 4) was associated with a faster transition to dementia.

### Regression Models

Hazard ratios estimated by multivariate Cox proportional hazard models are presented in Table 2. All models were adjusted for age, gender, tinnitus, and co-morbidities. The presence of bilateral hearing impairment increased the risk of an incident dementia diagnosis by 16% (HR = 1.16, p<0.001), while unilateral hearing impairment (HR = 0.80, p = 0.006) and other diseases of the ear (HR = 0.90, p<0.001) seem to decrease the risk (Model 1). The effects of unilateral hearing impairment and other diseases of the ear were no longer significant if treatment by an ENT doctor was taken into account. The effect of bilateral (HR = 1.51, p<0.001) and side-unspecified hearing impairment remained significant, implying that the effect of hearing impairment on dementia incidence is only partly mediated through ENT doctor utilization. Treatment by an ENT doctor itself decreased the risk of dementia incidence by 26% (HR = 0.74, p<0.001; Model 2). Hazard ratios for hearing impairment changed only marginally when we adjusted for care level, institutionalization, and depression, respectively (Models 3–5). The risk of incident dementia diagnosis increased with care level and was highest for those persons with care level 3 (HR = 8.26, p<0.001) compared to persons without care level, and was still 4.72 (p<0.001) for care level 1 and 6.62 (p<0.001) for care level 2 (Model 3). The risk of dementia was more than doubled for persons living in a nursing home (HR = 2.23, p<0.001; Model 4). Controlling for institutionalization decreased the hazard ratios for care level by roughly a quarter, while those for treatment by an ENT doctor remained unchanged. While controlling for all other information (Model 5) the risk of dementia for persons with a depression diagnosis increased by 36% (HR = 1.36; p<0.001).

**Table 2:** Hazard ratios of incident dementia by hearing impairment and key variables.

		Model 1		Model 2		Model 3		Model 4		Model 5	
Variable	Value	HR	p	HR	p	HR	p	HR	p	HR	p
Hearing impairment	no (reference)	1		1		1		1		1	
	unilateral	0.80	0.006	1.03	0.771	1.05	0.555	1.04	0.647	1.01	0.877
	bilateral	1.16	0.000	1.51	0.000	1.43	0.000	1.45	0.000	1.43	0.000
	side unspecified	0.97	0.265	1.23	0.000	1.21	0.000	1.22	0.000	1.20	0.000
	other diseases of the ear	0.90	0.000	1.01	0.853	1.01	0.650	1.02	0.561	1.00	0.890
Treatment by an ENT doctor	no (reference)			1		1		1		1	
	yes			0.74	0.000	0.82	0.000	0.82	0.000	0.81	0.000
Care level	no (reference)					1		1		1	
	1					4.72	0.000	4.09	0.000	3.98	0.000
	2					6.62	0.000	5.21	0.000	5.04	0.000
	3					8.26	0.000	6.13	0.000	5.96	0.000
Nursing home	no (reference)							1		1	
	yes							2.23	0.000	2.19	0.000
Depression	no (reference)									1	
	yes									1.36	0.000

Controlled for age, gender, tinnitus, and co-morbidities (see S2 Table); HR = hazard ratio; Data source: Claims data AOK 2006–2010; Hazard ratios of incident dementia by covariates presented in S2 Table

In interaction effects models, we found significant main effects for hearing impairment, particularly for bilateral and unspecified hearing impairment which increase the risk of dementia. We found a significant main effect for treatment by an ENT doctor which lowers the risk of dementia, while the main effects of care level, nursing home, and depression are associated with an increased risk. The statistically significant interaction effect below one (HR = 0.83, p = 0.004) for those with bilateral hearing impairment and professional treatment implies a comparatively lower dementia risk of the professionally treated as compared to those without ENT treatment (Table 3). There were also significant interaction effects below one for individuals with bilateral hearing impairment and care level 1 (HR = 0.75, p<0.001) or 2 (HR = 0.84, p = 0.003), as well as for hearing impairment with unspecified side and care level 1 (HR = 0.88, p = 0.022) or 2 (HR = 0.85, p = 0.021) (Table 4). Similarly, individuals with bilateral hearing impairment and who were institutionalized had an interaction effect below one (HR = 0.90, p = 0.43; Table 5). There was no significant interaction between hearing impairment and depression (Table 6).

**Table 3:** Interaction of hearing impairment and treatment by an ENT doctor.

		Model 6	
Variable	Value	HR	p
Hearing impairment	no	1	
	unilateral	0.91	0.638
	bilateral	1.57	0.000
	side unspecified	1.16	0.001
	other diseases of the ear	0.97	0.321
Treatment by an ENT doctor	no	1	
	yes	0.87	0.003
Hearing impairment ˣ Treatment by an ENT doctor	unilateral ˣ yes	1.06	0.799
	bilateral ˣ yes	0.83	0.004
	side unspecified ˣ yes	0.97	0.688
	other diseases of the ear ˣ yes	-*	

Controlled for all other information in Model 5, Table 2; HR = hazard ratio; Data source: Claims data AOK 2006–2010;

*omitted; Hazard ratios of incident dementia by covariates presented in S2 Table

**Table 4:** Interaction of hearing impairment and care level.

		Model 7	
Variable	Value	HR	p
Hearing impairment	no	1	
	unilateral	1.04	0.737
	bilateral	1.63	0.000
	side unspecified	1.29	0.000
	other diseases of the ear	1.03	0.497
Care level	no	1	
	1	4.38	0.000
	2	5.38	0.000
	3	6.09	0.000
Hearing impairment ˣ Care level	unilateral ˣ 1	1.02	0.935
	unilateral ˣ 2	0.96	0.872
	unilateral ˣ 3	0.92	0.850
	bilateral ˣ 1	0.75	0.000
	bilateral ˣ 2	0.84	0.003
	bilateral ˣ 3	0.87	0.217
	side unspecified ˣ 1	0.88	0.022
	side unspecified ˣ 2	0.85	0.021
	side unspecified ˣ 3	0.93	0.586
	other diseases of the ear ˣ 1	0.92	0.192
	other diseases of the ear ˣ 2	0.96	0.536
	other diseases of the ear ˣ 3	1.14	0.307

Controlled for all other information in Model 5, Table 2; HR = hazard ratio; Data source: Claims data AOK 2006–2010; Hazard ratios of incident dementia by covariates presented in S2 Table

**Table 5:** Interaction of hearing impairment and nursing home.

		Model 8	
Variable	Value	HR	p
Hearing impairment	no	1	
	unilateral	1.05	0.603
	bilateral	1.46	0.000
	side unspecified	1.21	0.000
	other diseases of the ear	0.99	0.834
Nursing home	no	1	
	yes	2.26	0.000
Hearing impairment ˣ Nursing home	unilateral ˣ yes	0.82	0.390
	bilateral ˣ yes	0.90	0.043
	side unspecified ˣ yes	0.96	0.565
	other diseases of the ear ˣ yes	1.02	0.723

Controlled for all other information in Model 5, Table 2; HR = hazard ratio; Data source: Claims data AOK 2006–2010; Hazard ratios of incident dementia by covariates presented in S2 Table

**Table 6:** Interaction of hearing impairment and depression.

		Model 9	
Variable	Value	HR	p
Hearing impairment	no	1	
	unilateral	1.02	0.883
	bilateral	1.45	0.000
	side unspecified	1.19	0.000
	other diseases of the ear	1.02	0.557
Depression	no	1	
	yes	1.38	0.000
Hearing impairment ˣ Depression	unilateral ˣ yes	0.99	0.940
	bilateral ˣ yes	0.96	0.381
	side unspecified ˣ yes	1.01	0.861
	other diseases of the ear ˣ yes	0.94	0.242

Controlled for all other information in Model 5, Table 2; HR = hazard ratio; Data source: Claims data AOK 2006–2010; Hazard ratios of incident dementia by covariates presented in S2 Table

## Discussion

Hearing impairment increases the risk of dementia. The effect is partly independent of treatment by an ENT doctor, care level, institutionalization, depression, and co-morbidities. This is not only in line with our previous study [12] and other studies [8, 10, 11, 40] on the association between hearing impairment and dementia; it is also in line with a series of studies on the particular impact of hearing impairment on dementia incidence. In a prospective study of 639 participants, Lin et al. [42] found an increase in the risk of all-cause dementia and Alzheimer’s disease depending on the severity of baseline hearing loss. Based on the results of their case control study, Uhlmann et al. [9] hypothesized that hearing impairment contributes to cognitive dysfunction.

Our focus is on hearing impairment, and according to the International Classification of Diseases we also take laterality as well as other diseases of the ear and mastoid process not related to hearing impairment into account. As expected we found significant effects for bilateral hearing impairment, i.e. an increased risk for dementia incidence. We also found an increased risk of dementia for persons with unspecified hearing impairment. We suppose that this effect is driven by underlying bilateral hearing impairment although the laterality is unspecified by the diagnosing physician. Controlling for all information in the full model (Model 5, Table 2), we found no effect for unilateral hearing impairment and for diseases of the ear that are not related to hearing impairment. In sensitivity analyses using only age-related and sensorineural hearing loss (presbycusis), we could not differentiate by laterality (results not shown). However, we did find results consistent with those presented above. The prevalence of age-related and sensorineural hearing loss is highest among all hearing impairment diagnoses included in our study and is thus supposed to be the main driver of the results.

**Fig 5:**
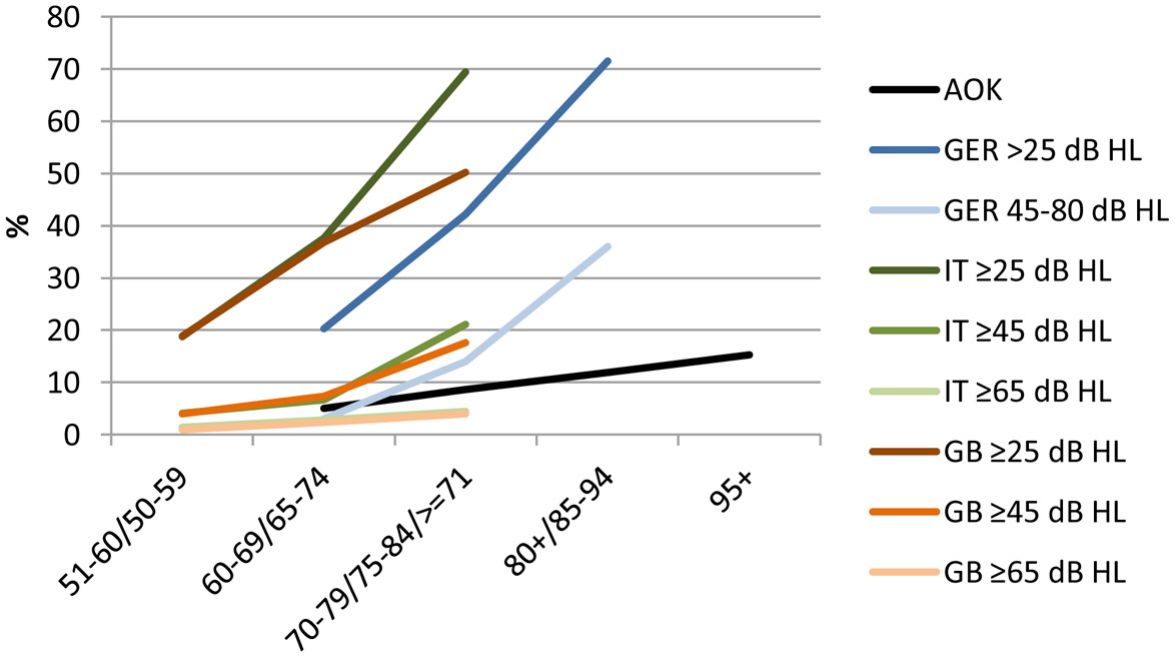
Age-specific prevalence of hearing impairment in health claims data and three epidemiologic studies. Sources: AOK: health claims data AOK 2006–2010, own calculations; GER—Germany [50]; IT—Italy [51]; GB—Great Britain [52]; dB HL = decibel hearing level.

In order to assess actual hearing ability, it is necessary to use formal audiometry measures, such as pure tone threshold average, speech discrimination scores, or word recognition scores [43]. Whilst a standardized format for reporting hearing ability based on such measures, e.g. in clinical trials [43], would be preferable, such precise information is not always available. Multiple studies have used subjective reports from patients, family, or caregivers, as well as interviewer assessment of hearing status (e.g. [6, 44, 45]), while the validity and specificity of self-reported hearing impairment has been proven elsewhere [46–48]. Using formal diagnoses from health claims data is another option for assessing hearing impairment on a population-based level [49]. One limitation of using health claims data is the lack of information on assessment and severity of hearing impairment. We therefore compared the prevalence of hearing impairment in claims data to three national and international epidemiologic studies on hearing status [50–52] and found similar prevalence estimations related to moderate and severe impairment (Fig 5). According to the World Health Organization, a hearing level between 41–60 dB is defined as moderate impairment. Affected individuals are able to hear and repeat words spoken in raised voice at 1 meter and are usually recommended to use hearing aids. With a hearing level between 61–80 dB, which is defined as severe impairment, individuals are able to hear some words shouted into the stronger ear. They are recommended to use hearing aids, or lip-reading and signing should be taught [53]. Although we still do not know the actual severity, this comparison permits us to assume that the diagnoses in claims data are predominantly related to persons who receive treatment because of hearing impairment of moderate and severe nature. This may partly account for the high effect size of the impact of hearing impairment on dementia onset.

We adjusted for a series of information to obtain potential mediating or moderating effects on the pathway between hearing impairment and dementia. One important aspect in the analysis of hearing impairment and dementia is the use of hearing aids. Hearing aids may delay or prevent the occurrence of dementia by improving the ability of hearing. Studies found a positive association of hearing aid use and cognition [40]. Unfortunately, information on hearing aids is not available in the current claims data. Instead we use treatment by an ENT doctor as a potential proxy variable. In Germany, the use of assistive technologies such as hearing or visual aids is regulated by resource policies. According to these policies, the prescription of hearing aids depends on several requirements and the first prescription is bound to evaluation and treatment given by an ENT doctor [54]. The benefit of hearing aids obviously depends not only on the prescription thereof, but also on their actual use. However, the sporadic or non-use is common among the elderly [55, 56]. The effect of hearing impairment on dementia incidence is only partly mediated and remains significant when we adjust for this information. The effect size of treatment by an ENT doctor is not affected when we introduce institutionalization and depression. This is in line with the findings of Dawes et al. [40] who suggested two explanations. First, any positive effects of hearing aid use on cognition may be via improvement in hearing ability or associated increases in self-efficacy. Second, positive associations between hearing aid use and cognition may be accounted for by more cognitively able people seeking and using hearing aids. The positive effect of treatment by a specialist for diseases of the ear found in our data may work similarly. On the one hand, treatment by an ENT doctor is associated with adequate treatment and possibly with hearing aid use, subsequently leading to improved hearing ability. On the other hand, persons with better cognition are more likely to be aware of one’s own hearing impairment and are more likely to seek treatment.

We included care level to account for the general frailty and vulnerability of older persons and found an increased risk for dementia with higher care level. Findings on the association between frailty and dementia are somewhat inconsistent with regard to type of dementia. Gray et al. [57] found evidence for an association between frailty and non-AD dementia, but not with AD. An association with all-cause dementia was found only in participants with higher cognitive scores at baseline. In contrast, Buchman et al. [58] found an association between frailty and incidence of Alzheimer’s disease. The interaction with hearing impairment points towards a decreased risk of dementia for the bilateral and side-unspecified hearing impaired with care level 1 and 2. We suggest two possible explanations. First, individuals benefit from being under observation during the assessment process for assignment to care level where a hearing impairment can be recognized and treated. Second, there may be a selection effect. It may be more complicated for individuals with hearing impairment to organize everything necessary for the process of being assigned a care level. Thus, people with hearing impairment who receive a care level may be selected by social and health characteristics.

Our results reveal that living in a nursing home had a direct effect on the transition to dementia but does not decrease the effect size of hearing impairment. Thus, institutionalization does not work as a mediator in our study. With regard to the significant interaction effect below one for the individuals with bilateral hearing impairment and who were institutionalized, institutionalization rather works as a moderator in our study. We suggest that individuals living in a nursing home benefit from being under daily observation, and that an occurring or existing hearing impairment is more likely to be noticed and treated, subsequently leading to a decreased risk for dementia.

Another important aspect is depression. Depression is associated with hearing impairment [59]; it is both a risk factor and a prodrome of Alzheimer’s disease and is a common occurrence in all types of dementias as well as in mild cognitive impairment [60]. In our study, depression had neither a mediating nor a moderating effect on the pathway between hearing impairment and dementia incidence, but had itself an effect of incident dementia diagnosis.

A high number of co-morbidities increased the risk of dementia incidence. The indicator used in our study includes several diseases known as risk factors for cognitive decline and dementia, e.g. vascular diseases [61–64], cerebrovascular diseases [65, 66], or impairment of (lower) extremities [67, 68]. Using the single diseases instead of the combined indicator did not change the overall results of hearing impairment and other variables of interest.

Although we controlled for a series of relevant information, there remained a direct effect of hearing impairment on dementia. Several possible mechanisms may explain this effect. First, a common neurodegenerative process may induce hearing impairment as well as cognitive impairment through decreased functionality of the auditory system [39] and of the higher-order association cortex [38], both known as the neuronal basis for selective attention to auditory stimuli [69]. Thus, neurodegeneration may lead to a dysfunction of the central auditory system, which was found to be associated with Alzheimer’s diseases as the most common type of dementia [70]. Second, both hearing impairment and dementia are caused by a neurobiological process such as a vascular disease. We controlled for vascular and cerebrovascular diseases in our regression models and still found a significant effect of hearing impairment on dementia. Analogous to the studies of Lin et al. [42, 71], excluding patients with a history of cerebrovascular diseases (n = 29,425; including stroke and transient ischemic attack) during the observation period did not substantially change the main findings (results not shown). Third, in the course of a pathophysiologic interaction between hearing impairment and neurodegeneration, a co-occurring sensory deprivation and overproduction of the amyloid precursor protein may lead to dendritic shrinkage, subsequently affecting the neuronal transmission of information [72]. Fourth, according to the concept of cognitive reserve, people with a high reserve are more able to compensate for a decline in brain functions than people with a low reserve [73]. The reallocation of processing resources to compensate for a deterioration in the auditory system may be detrimental to cognitive resources such as working memory [32], subsequently leading to a faster cognitive decline. Fifth, results of cognition tests may be confounded by hearing impairment. The confounding effect seems to be inconsistent and was shown for tests of episodic and semantic long-term memory but not for tests of short-term memory [74]. Sixth, the results may be sensitive to physicians’ awareness of diseases. Physicians who are more likely to diagnose hearing impairment may also be more likely to diagnose dementia and vice versa. This might be true if any diagnosis of hearing impairment regardless of laterality is associated with a dementia diagnosis. In the claims data, only bilateral hearing impairment should be associated with a higher dementia incidence, which was the case in the current study. No effect was found for unilateral hearing impairment on dementia incidence.

We have to consider potential limitations associated with the data. Other studies analyzed the specific impact of hearing impairment on Alzheimer’s disease or dementia of the Alzheimer’s type [49, 75, 76]. However, the current data do not allow for analyzing such specific diagnoses because the different subtypes of dementia cannot be meaningfully distinguished. According to findings of epidemiological studies, Alzheimer’s disease is the most [70, 77] and vascular dementia the second most prevalent cause of dementia [77, 78]. In the AOK data, only 27% of dementia cases were diagnoses of Alzheimer’s disease but 45–50% of the dementia diagnoses were of unspecified dementia [41]. Health claims data contain diagnoses from all medical doctors, including from general practitioners. In contrast to epidemiological studies, which use face-to-face examinations following defined protocols and are performed by neurologists or psychiatrists, dementia diagnoses in medical claims data are neither specific nor standardized. Nonetheless, dementia prevalence and incidence based on AOK claims data fit well to national and international studies [1, 20].

Health claims data are primarily relevant for cost reimbursement and cost calculation, which leads to two issues. First, only those diagnoses leading to treatment are relevant for the purposes of cost calculation. Thus, a patient’s cognitive impairment might not be documented if no further treatment is given. This could be particularly true for mild cases of dementia and cognitive impairment. The incidence of dementia will certainly be biased to higher ages when the symptoms of the disease become more obvious [20]. Second, many important aspects with regard to dementia are not relevant for cost reimbursement and are thus not collected. The impacts of genetic factors [79], education and socioeconomic status [80, 81], or life style factors such as physical activity [82, 83], nutrition [84, 85], alcohol consumption [64, 86], and smoking [87, 88] have been extensively examined in the literature. However, such information is not available in the current data.

The strength of this study is the large longitudinal sample with more than 150,000 persons including the institutionalized. Excluding the institutionalized population would lead to a bias, as dementia incidence is four times higher among those living in nursing homes or assisted living compared to those elder who do not live in such facilities [89]. Similar, the incidence of hearing loss in institutionalized residents is higher compared to community-based residents [90]. There is no bias in the results due to self-selection, selection by the health care provider, or the study design. Using medical diagnoses also prevents recall bias by the patient.

## Conclusions

Hearing impairment increases the risk of dementia incidence, underlining the importance of good hearing ability. A screening for hearing impairment should be considered in case of cognitive impairment [91], and a wider screening for dementia in case of proven hearing impairment and clinical suspicion of cognitive impairment [30]. Preserving hearing ability and providing early treatment of hearing impairment may maintain social participation and may reduce the burden associated with dementia. Although formal audiometric measures are preferable and subjective reports of hearing ability are often used, health claims data provide an alternative to assessing hearing ability on a population-based level. Our results should be confirmed in additional studies with a special focus on the impact of hearing aid which offers potential intervention on the pathway to dementia.

## Supporting Information

S1 Table Dementia incidence rates by covariates. Data source: Claims data AOK 2006–2010. IR: Incidence rate per 100 PY. LCI: 95% lover confidence interval. UCI: 95% upper confidence interval.
(DOCX)

S2 Table Hazard ratios of incident dementia by covariates and key variables. S2 Table presents the effects of gender, age, comorbidities, tinnitus, treatment by an ENT doctor, care level, nursing home, and depression, respectively, not shown in Tables 2–6 in main text; Data source: Claims data AOK 2006–2010.
(DOCX)
