# Supplementary material for: Hearing Impairment Affects Dementia Incidence. An Analysis Based on Longitudinal Health Claims Data in Germany
Source: PLoS One. 2016 Jul 8;11(7):e0156876. doi: 10.1371/journal.pone.0156876 (PMC4938406; doi:10.1371/journal.pone.0156876)
Supplement: S1 Table — Data source: Claims data AOK 2006–2010. IR: Incidence rate per 100 PY. LCI: 95% lover confidence interval. UCI: 95% upper confidence interval. (DOCX) [file pone.0156876.s001.docx]

**S1 Table. Dementia incidence rates by covariates**

| **Variable** | **Value** | **Person-years (PY)** | **Cases** | **IR** | **LCI** | **UCI** |
| --- | --- | --- | --- | --- | --- | --- |
| Gender | male | 270874 | 4758 | 1.76 | 1.71 | 1.81 |
|  | female | 398650 | 9844 | 2.47 | 2.42 | 2.52 |
| Age | 65-69 | 226623 | 994 | 0.44 | 0.41 | 0.47 |
|  | 70-74 | 171544 | 1826 | 1.06 | 1.02 | 1.11 |
|  | 75-79 | 129075 | 2979 | 2.31 | 2.23 | 2.39 |
|  | 80-84 | 85998 | 3787 | 4.40 | 4.27 | 4.55 |
|  | 85-89 | 41572 | 3164 | 7.61 | 7.35 | 7.88 |
|  | 90-94 | 11205 | 1318 | 11.76 | 11.14 | 12.41 |
|  | 95+ | 3507 | 534 | 15.23 | 13.99 | 16.57 |
| Comorbidities | 0 | 54297 | 420 | 0.77 | 0.70 | 0.85 |
|  | 1 | 98772 | 909 | 0.92 | 0.86 | 0.98 |
|  | 2 | 147639 | 1941 | 1.31 | 1.26 | 1.37 |
|  | 3 | 145984 | 2896 | 1.98 | 1.91 | 2.06 |
|  | 4 | 109329 | 3175 | 2.90 | 2.80 | 3.01 |
|  | 5 | 66042 | 2599 | 3.94 | 3.79 | 4.09 |
|  | 6 | 31777 | 1631 | 5.13 | 4.89 | 5.39 |
|  | 7 or more1 | 15684 | 1031 | 6.57 | 6.18 | 6.99 |
| Tinnitus | no | 600942 | 13385 | 2.23 | 2.19 | 2.27 |
|  | yes | 68581 | 1217 | 1.77 | 1.68 | 1.88 |
| Total |  | 669524 | 14602 | 2.18 | 2.15 | 2.22 |

Data source: Claims data AOK 2006-2010

IR: Incidence rate per 100 PY

LCI: 95% lover confidence interval

UCI: 95% upper confidence interval
