# Supplementary material for: Hearing Impairment Affects Dementia Incidence. An Analysis Based on Longitudinal Health Claims Data in Germany
Source: PLoS One. 2016 Jul 8;11(7):e0156876. doi: 10.1371/journal.pone.0156876 (PMC4938406; doi:10.1371/journal.pone.0156876)
Supplement: S2 Table — presents the effects of gender, age, comorbidities, tinnitus, treatment by an ENT doctor, care level, nursing home, and depression, respectively, not shown in Tables 2–6 in main text; Data source: Claims data AOK 2006–2010. (DOCX) [file pone.0156876.s002.docx]

**S2 Table. Hazard ratios of incident dementia by covariates and key variables**

|  |  | **Model 1** | | **Model 2** | | **Model 3** | | **Model 4** | | **Model 5** | | **Model 6** | | **Model 7** | | **Model 8** | | **Model 9** | |
| --- | --- | --- | --- | --- | --- | --- | --- | --- | --- | --- | --- | --- | --- | --- | --- | --- | --- | --- | --- |
| **Variable** |  | **HR** | **p** | **HR** | **p** | **HR** | **p** | **HR** | **p** | **HR** | **p** | **HR** | **p** | **HR** | **p** | **HR** | **p** | **HR** | **p** |
| Gender | male (reference) | 1 |  | 1 |  | 1 |  | 1 |  | 1 |  | 1 |  | 1 |  | 1 |  | 1 |  |
|  | female | 1.00 | 0.818 | 1.00 | 0.827 | 0.93 | <0.001 | 0.91 | <0.001 | 0.86 | <0.001 | 0.87 | <0.001 | 0.86 | <0.001 | 0.86 | <0.001 | 0.86 | <0.001 |
| Age | 65-69 | 1 |  | 1 |  | 1 |  | 1 |  | 1 |  | 1 |  | 1 |  | 1 |  | 1 |  |
|  | 70-74 | 2.37 | <0.001 | 2.36 | <0.001 | 2.29 | <0.001 | 2.30 | <0.001 | 2.32 | <0.001 | 2.32 | <0.001 | 2.31 | <0.001 | 2.32 | <0.001 | 2.32 | <0.001 |
|  | 75-79 | 4.68 | <0.001 | 4.66 | <0.001 | 4.17 | <0.001 | 4.21 | <0.001 | 4.26 | <0.001 | 4.27 | <0.001 | 4.23 | <0.001 | 4.26 | <0.001 | 4.26 | <0.001 |
|  | 80-84 | 8.36 | <0.001 | 8.30 | <0.001 | 6.26 | <0.001 | 6.27 | <0.001 | 6.39 | <0.001 | 6.40 | <0.001 | 6.32 | <0.001 | 6.37 | <0.001 | 6.39 | <0.001 |
|  | 85-89 | 13.83 | <0.001 | 13.68 | <0.001 | 7.81 | <0.001 | 7.63 | <0.001 | 7.84 | <0.001 | 7.84 | <0.001 | 7.79 | <0.001 | 7.82 | <0.001 | 7.84 | <0.001 |
|  | 90-94 | 20.46 | <0.001 | 20.08 | <0.001 | 8.24 | <0.001 | 7.58 | <0.001 | 7.95 | <0.001 | 7.95 | <0.001 | 7.93 | <0.001 | 7.95 | <0.001 | 7.95 | <0.001 |
|  | 95+ | 27.42 | <0.001 | 26.50 | <0.001 | 7.94 | <0.001 | 6.93 | <0.001 | 7.40 | <0.001 | 7.39 | <0.001 | 7.39 | <0.001 | 7.41 | <0.001 | 7.40 | <0.001 |
| Comorbidities | 0 (reference) | 1 |  | 1 |  | 1 |  | 1 |  | 1 |  | 1 |  | 1 |  | 1 |  | 1 |  |
|  | 1 | 1.08 | 0.192 | 1.08 | 0.190 | 1.03 | 0.664 | 1.03 | 0.620 | 1.00 | 0.952 | 1.00 | 0.943 | 0.99 | 0.894 | 1.00 | 0.970 | 1.00 | 0.983 |
|  | 2 | 1.37 | <0.001 | 1.37 | <0.001 | 1.24 | <0.001 | 1.24 | <0.001 | 1.20 | 0.001 | 1.20 | 0.001 | 1.18 | 0.002 | 1.20 | 0.001 | 1.20 | 0.001 |
|  | 3 | 1.85 | <0.001 | 1.85 | <0.001 | 1.50 | <0.001 | 1.51 | <0.001 | 1.44 | <0.001 | 1.44 | <0.001 | 1.41 | <0.001 | 1.43 | <0.001 | 1.43 | <0.001 |
|  | 4 | 2.46 | <0.001 | 2.45 | <0.001 | 1.74 | <0.001 | 1.74 | <0.001 | 1.63 | <0.001 | 1.63 | <0.001 | 1.61 | <0.001 | 1.63 | <0.001 | 1.63 | <0.001 |
|  | 5 | 3.16 | <0.001 | 3.15 | <0.001 | 1.96 | <0.001 | 1.96 | <0.001 | 1.82 | <0.001 | 1.82 | <0.001 | 1.79 | <0.001 | 1.82 | <0.001 | 1.82 | <0.001 |
|  | 6 | 3.87 | <0.001 | 3.86 | <0.001 | 2.12 | <0.001 | 2.10 | <0.001 | 1.93 | <0.001 | 1.92 | <0.001 | 1.90 | <0.001 | 1.92 | <0.001 | 1.92 | <0.001 |
|  | 7 or more | 4.98 | <0.001 | 4.96 | <0.001 | 2.33 | <0.001 | 2.27 | <0.001 | 2.06 | <0.001 | 2.06 | <0.001 | 2.03 | <0.001 | 2.05 | <0.001 | 2.05 | <0.001 |
| Tinnitus | no (reference) | 1 |  | 1 |  | 1 |  | 1 |  | 1 |  | 1 |  | 1 |  | 1 |  | 1 |  |
|  | yes | 0.82 | <0.001 | 0.84 | <0.001 | 0.95 | 0.142 | 0.97 | 0.277 | 0.95 | 0.098 | 0.95 | 0.125 | 0.94 | 0.048 | 0.95 | 0.081 | 0.95 | 0.100 |
| Treatment by an ENT doctor | no (reference) |  |  |  |  |  |  |  |  |  |  |  |  | 1 |  | 1 |  | 1 |  |
|  | yes |  |  |  |  |  |  |  |  |  |  |  |  | 0.81 | <0.001 | 0.81 | <0.001 | 0.81 | <0.001 |
| Care level | no (reference) |  |  |  |  |  |  |  |  |  |  | 1 |  |  |  | 1 |  | 1 |  |
|  | 1 |  |  |  |  |  |  |  |  |  |  | 3.97 | <0.001 |  |  | 3.98 | <0.001 | 3.98 | <0.001 |
|  | 2 |  |  |  |  |  |  |  |  |  |  | 5.04 | <0.001 |  |  | 5.04 | <0.001 | 5.04 | <0.001 |
|  | 3 |  |  |  |  |  |  |  |  |  |  | 5.94 | <0.001 |  |  | 5.94 | <0.001 | 5.96 | <0.001 |
| Nursing home | no (reference) |  |  |  |  |  |  |  |  |  |  | 1 |  | 1 |  |  |  | 1 |  |
|  | yes |  |  |  |  |  |  |  |  |  |  | 2.19 | <0.001 | 2.19 | <0.001 |  |  | 2.19 | <0.001 |
| Depression | no (reference) |  |  |  |  |  |  |  |  |  |  | 1 |  | 1 |  | 1 |  |  |  |
|  | yes |  |  |  |  |  |  |  |  |  |  | 1.36 | <0.001 | 1.36 | <0.001 | 1.36 | <0.001 |  |  |

Table S2 presents the effects of gender, age, comorbidities, tinnitus, treatment by an ENT doctor, care level, nursing home, and depression, respectively, not shown in Table 2-6 in main text; Data source: Claims data AOK 2006-2010
